# Evidence that Altered *Cis* Element Spacing Affects PpsR Mediated Redox Control of Photosynthesis Gene Expression in *Rubrivivax gelatinosus*

**DOI:** 10.1371/journal.pone.0128446

**Published:** 2015-06-01

**Authors:** Takayuki Shimizu, Zhuo Cheng, Katsumi Matsuura, Shinji Masuda, Carl E. Bauer

**Affiliations:** 1 Department of Biological Sciences, Tokyo Institute of Technology, Kanagawa 226–8501, Japan; 2 Department of Molecular and Cellar Biochemistry, Indiana University, Bloomington, Indiana 47405, United States of America; 3 Graduate School of Science and Engineering, Tokyo Metropolitan University, Tokyo 192–0397, Japan; 4 Center for Biological Resources and Informatics, Tokyo Institute of Technology, Kanagawa 226–8501, Japan; 5 Earth-Life Science Institute, Tokyo Institute of Technology, Tokyo 152–8551, Japan; University of Iowa, UNITED STATES

## Abstract

PpsR is a major regulator of photosynthesis gene expression among all characterized purple photosynthetic bacteria. This transcription regulator has been extensively characterized in *Rhodobacter* (*Rba*.) *capsulatus* and *Rba*. *sphaeroides* which are members of the α-proteobacteria lineage. In this study, we have investigated the biochemical properties and mutational effects of a *ppsR* deletion strain in the β-proteobacterium *Rubrivivax* (*Rvi*.) *gelatinosus* in order to reveal phylogenetically conserved mechanisms and species-specific characteristics. A deletion of the *ppsR* gene resulted in de-repression of photosystem synthesis showing that PpsR functions as a repressor of photosynthesis genes in this species. We also constructed a *Rvi*. *gelatinosus* PpsR mutant in which a conserved cysteine at position 436 was changed to an alanine to examine whether or not this residue is important for sensing redox, as reported in *Rhodobacter* species. Surprisingly, the Cys436 Ala mutant retained the ability to repress photosynthesis gene expression under aerobic conditions, suggesting that PpsR from *Rvi*. *gelatinosus* has different redox-responding characteristics. Furthermore, biochemical analyses demonstrated that *Rvi*. *gelatinosus* PpsR only shows redox-dependent binding to promoters with 9-bp spacing, but not 8-bp spacing, between two PpsR-recognition sequences. These results indicate that redox-dependent binding of PpsR requires appropriate *cis* configuration of PpsR target sequences in *Rvi*. *gelatinosus*. These results also indicate that PpsR homologs from different species regulate photosynthesis genes with altered biochemical properties.

## Introduction

Many purple photosynthetic bacteria can grow either using aerobic respiration or anaerobic photosynthesis. These cells tightly control synthesis of their photosynthetic apparatus in response to oxygen tension and light intensity [1, 2]. Under aerobic conditions, light-excited bacteriochlorophylls are capable of generating reactive oxygen species (ROS) such as singlet oxygen as a side reaction. ROS are very damaging to cells as they react with many biomolecules including DNA [3]. In order to avoid the production of ROS, most purple photosynthetic bacteria repress synthesis of their photosynthetic apparatus immediately after being exposed to oxygen. One of the main factors responsible for this regulation is the transcription factor PpsR which is universally present among purple photosynthetic bacteria (in some species PpsR is also called CrtJ) [4, 5]. However, PpsR/CrtJ homologes from different purple photosynthetic bacterial species only exhibit 26–54% sequence identity indicating that their properties may exhibit variations [6].

Previous studies have demonstrated that PpsR binds to the consensus sequence (TGT-N_12_-ACA) that is present in many photosynthesis gene promoters including those involved in controlling synthesis of carotenoids, bacterichlorophyll and structural light harvesting proteins. PpsR is known to be a redox-responding repressor as it has a higher affinity for target DNA sequences under oxidizing conditions than under reducing conditions in *Rba*. *capsulatus* and *Rba*. *sphaeroides* [7, 8]. This change of binding affinity is thought to be caused by formation of an intramolecular disulfide bond between two cysteine residues (Cys249 and Cys420 in *Rba*. *capsulatus*) and/or oxidative modification of the latter cysteine (Cys420) to a sulfenic acid derivative [8, 9]. Site-directed mutation of Cys420 in *Rba*. *capsulatus* CrtJ or Cys424 in *Rba*. *sphaeroides* PpsR resulted in a significant decrease of DNA binding activity of the repressor *in vitro* [9, 10]. Additional expression studies showed that a Cys420 to alanine mutation (C420A) caused elevated aerobic expression of photosynthesis genes to the same level as did a *crtJ/ppsR* disrupted strain of *Rba*. *capsulatus* [9]. This is in contrast to C249A mutation that only partially de-repressed aerobic photosystem expression indicating that the oxidation state of Cys420 is key to redox regulation of PpsR/CrtJ in the tested *Rhodobacter* species [9].

An interesting divergence occurs in a photosynthetic *Bradyrhizobium* strain that has two *ppsR* genes, PpsR1 and PpsR2. Unlike the *Rba*. *capsulatus and Rba*. *sphaeroides* homologs, the DNA binding affinity of *Bradyrhizobium* PpsR1 is reported to be higher under reducing conditions than under oxidizing conditions. Furthermore PpsR1 contains only one cysteine (Cys429) and this cysteine alone is sufficient for sensing redox [11]. Interestingly, PpsR2 does not contain a similar cysteine in the HTH motif and is thought to be dedicated to light control of photosynthesis gene expression rather than redox control [11]. Finally, PpsR2 from *Rhodopseudomonas palustris* also lacks a cysteine residue in the HTH motif [12]. Collectively, these observations suggest that there are species-specific alterations in redox-sensing mechanisms employed by PpsR.

To date, most research on PpsR/CrtJ has been confined with members of the α-proteobacteria lineage and, thus the generality of PpsR functionality among purple photosynthetic bacteria is not clear [6, 12]. In this study, we examined regulatory characteristics of PpsR from the β-proteobacterium *Rvi*. *gelatinosus* which exhibits only 33% sequence identity to *Rba*. *capsulatus* CrtJ. As is the case with *Rhodobacter* PpsR homologs, *Rvi*. *gelatinosus* PpsR has three cysteine residues with one Cys436 located in the HTH motif. In this study we investigated biochemical and physiological properties of wild type and the C436A mutant PpsR *in vitro* and *in vivo* and have highlight dissimilarities that exist from properties of that have been reported for PpsR homologs from other species.

## Materials and Methods

### Bacterial strains, media, and growth conditions

*Escherichia* (*E*.) *coli* strains JM109/*λpir*, S17-1/*λpir* and BL21 (DE3) were used for cloning, conjugal transfer of plasmids, and protein overexpression, respectively. *E*. *coli* cells were routinely grown in Luria Bertani (LB) medium at 37°C. Kanamycin was used at 50 μg/ml in LB medium.

*Rvi*. *gelatinosus* strain IL144 [13] and mutant strains were grown in PYS medium [14] under aerobic-dark (aerobically) or anaerobic-light (photosynthetically) condition at 30°C. Tetracyclin and kanamycin were used at 50 μg/ml in PYS medium.

### Cloning and mutagenesis of ppsR

Two 500-bp DNA fragments consisted of N-terminal and C-terminal regions of PpsR were amplified by polymerase chain amplifying (PCR) using KOD-plus- polymerase (TOYOBO). Two sets of primers were used for amplification. One set contained forward primer ppsRxbaIfor (5’-GGTCTAGAGCACTGCTGCTGCCGGC-3’) and the reverse primer ppsRsmaIrev (5’-TTTTTCCCGGGTCCAAACGGTCTCATTCGGA-3’). The other set contained forward primer ppsRsmaIfor (5’-TTTTTCCCGGGGAAGCCGAGAAGTAGAGCG-3’) and the reverse primer ppsRsacIrev (5’-GGGAGCTCCCAGCGACTTCAGCATCTAC-3’). *Xba*I, *Sma*I, *Sac*I and restriction sites were designed at additional polynucleotide tails present in the ppsRxbaIfor, ppsRsmaIrev and ppsRsmaIfor, and ppsRsacIrev primers, respectively (underlined). The first PCR fragment was digested with *Xba*I and *Sma*I and the second PCR fragment was digested with *Sac*I and *Sma*I. After digestion, these two fragments were mixed and ligated together into *Xba*I-*Sac*I-cut pJPCm [15]. A DNA cassette containing selectable Km^r^-marker as well as *sacB-sacR* genes of *Bacillus subtilis* was then inserted into the plasmid at the outside of the cloned fragment. The plasmid obtained called pJPΔ*ppsR*-KmSac was introduced into *Rvi*. *gelatinosus* cells by conjugation with the mobilizing *E*. *coli* strain S17-1/*λpir*. Cells undergoing single crossover events were selected by plating exconjugants on PYS plates containing kanamycin and tetracycline. After sequential cultivation in the absence of drug selection, sucrose-resistant but kanamycin-sensitive cells were selected on PYS-agar plates containing 15% sucrose to generate Δ*ppsR* mutant. A deletion of *ppsR* was confirmed by PCR amplification followed by sequence analysis. For Cys mutant construction, a *ppsR* clone containing 500-bp of DNA up- and down-stream of the gene was cloned into pJPCm using ppsRxbaIfor and ppsRsacIrev primers. A Cys436 to Ala mutation was then constructed using a QuickChange mutagenesis kit. The resulting plasmid pJPΔ*ppsR*C436A was then introduced into *Rvi*. *gelatinosus* Δ*ppsR* mutant cells with subsequent double crossover recombination event generating a C436A mutant variant of *ppsR*.

### Membrane preparation and spectrophotometric measurements

Wild-type and mutant strains of *Rvi*. *gelatinosus* were grown aerobically and photosynthetically to mid-log phase in PYS medium. Membrane fractions were prepared by French press cell disruption in 5 mM MES buffer (pH 7.0) containing 1 mM MgCl_2_, followed by ultracentrifugation for 20 min at 250,000 x*g*. The pelleted membranes were suspended and diluted with 5 mM MES buffer (pH 7.0) to a protein concentration of 100 μg protein/ml as measured by a Protein Assay kit (BioRad). Absorption spectra of membranes were recorded from 300 nm to 1000 nm using a UV-1800 spectrophotometer (SHIMADZU).

### RNA isolation, quantitative real-time PCR (QRT-PCR)

Wild-type and mutant strains of *Rvi*. *gelatinosus* were grown aerobically and photosynthetically to mid-log phase in PYS medium. 1 ml of cells were harvested with total RNA of each sample extracted using SV Total RNA Isolation System (Promega). A typical OD_260_ to OD_280_ ratio of RNA sample was approximately 2.0.

Reverse transcription was performed using a QuantiTect Reverse Transcription kit (QIAGEN). cDNA was amplified using SYBR Premix Ex Taq (TaKaRa). Signal detection and quantification were performed in duplicate using the Thermal Cycler Dice Real Time System (TaKaRa). Expression data were analyzed using 2^-ddCT^ formula [16]. As an internal control, the house-keeping gene *rpoZ* that encodes DNA-directed RNA polymerase omega subunit was used with the following gene-specific primers:

rpoZfor: 5’-GTCGAAGACTGCCTGCACAA-3’

rpoZrev: 5’-CCGGGCTTGTTCTTGGTCT-3’

pucBfor: 5’-GCCTGACCACGGCTGAAG-3’

pucBrev: 5’-ATACGGGTGCCATCAACCA-3’

crtIfor: 5’-CGACACCGGCTACCTCTACAA-3’

crtIrev: 5’-GTGCCGAAGTACCAGACGAAC-3’

### Overexpression and purification of PpsR and PpsR mutant

A PpsR overexpression vector was constructed by PCR amplification of the PpsR coding sequence using a forward primer containing an *Nde*I restriction site (5’-GGGGGGCATATGAGACCGTTTGGAGCACC-3’), and a reverse primer containing an *Hin*dIII restriction site (5’-TTTAAGCTTCTACTTCTCGGCTTCGGCC-3’). The PCR-amplified DNA segment was cloned into *Nde*I and *Hind*III sites in the pSUMO vector (LifeSensors Inc.) to generate the plasmid, pSUMO::PpsR. A Cys mutation in the pSUMO::PpsR construct was subsequently constructed using a QuickChange kit. pSUMO::PpsR was transformed into *E*. *coli* strain BL21 (DE3) and the recombinant protein SUMO-PpsR was overexpressed by induction of a 500 ml culture with 0.2 mM isopropyl-β-D-thiogalactopyranoside (IPTG) at 16°C overnight (12–16 h). Cells were harvested and resuspended in 20 ml nickel column loading buffer composed of 20 mM Tris-HCl (pH 8.0), 150 mM NaCl, 5 mM imidazole and 10% glycerol and lysed by two passes through a French pressure cell operated at 18,000 psi. The lysate was clarified by centrifugation at 30,000 x*g* for 30 min at 4°C. The resultant supernatant was passed through a 45 μm membrane filter (Millipore) and loaded onto a 1-ml HisTrap column (GE) with an ÄKTApurifier (GE), washed with 30-column-volume of wash buffer containing 20 mM Tris-HCl (pH 8.0), 150 mM NaCl, 20 mM imidazole, and 10% glycerol. SUMO-PpsR was eluted with a gradient of 20 mM imidazole to 500 mM imidazole in the loading buffer over a 15-column-volume total. The SUMO tag was then proteolytically cleaved at room temperature for 1 hour at a 100:1 molar ratio of UlP1 protease. Tag-less PpsR was then isolated by Superose 12 size exclusion chromatography in a PpsR standard buffer containing 20 mM Tris-HCl (pH 8.0), 500 mM NaCl, and 6% glycerol at a rate of 0.5 ml/min [8]. Peak fractions were analyzed by SDS-PAGE and PpsR containing fractions were concentrated. For generating the molecular weight standard curve by the size exclusion chromatography, Gel Filtration Standard (BioRad) was used as protein standard.

### Gel mobility shift analysis

**Fig 1:**
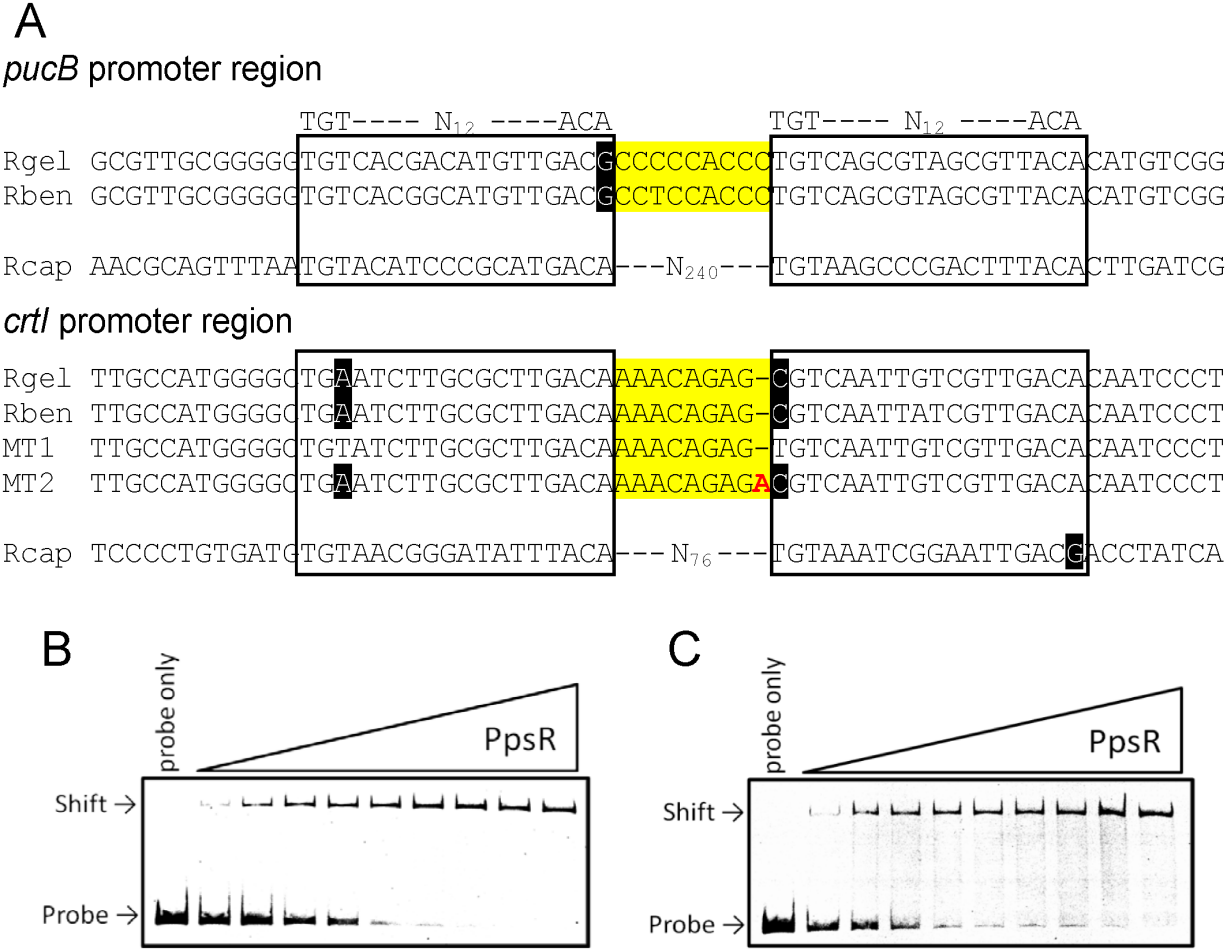
Sequence alignment and biochemical properties of the *pucB* and *crtI* promoter regions. (A) DNA-sequence alignments of the *pucB* and *crtI* promoter regions in *Rvi*. *gelatinosus* (Rgel), *Rvi*. *benzoatilyticus* strain JA2T (Rben) and *Rba*. *capsulatus* strain SB1003 (Rcap). In the *crtI* promoter regions of *Rvi*. *gelatinosus*, PpsR binding motif is changed to TGT-N_12_-ACA (MT1) and the space between the two PpsR binding sites is changed to 9-bp from 8-bp (MT2). An additional nucleotide is showed in red. PpsR binding sites and spaces between the two PpsR-binding sites are boxed and shown in yellow background, respectively. A different portion on the typical PpsR-binding sequence TGT-N_12_-ACA is indicated by black background. (B) Gel mobility shift assay using DNA fragment of *pucB* promoter regions under oxidizing conditions using from 15 nM to 180 nM PpsR. (C) Gel mobility shift assay using DNA fragment of *crtI* promoter regions under oxidizing conditions using from 45 nM to 210 nM PpsR.

Cy5-labeled DNA probes containing either the *pucB*, *crtI* promoter region were prepared by PCR amplification. 194-bp *pucB* promoter and 200-bp *crtI* promoter regions containing two TGT-N_12_-ACA PpsR-binding palindromes were amplified using primers pucBecoIfor (5’-GGGAATTCAGAGCTCCCGAGGCCCGCCG-3’) and pucBhindIIIrev (5’-GGAAGCTTACCAGTTTCCGTGCTCGACCC-3’), primers crtIecoIfor (5’-GGGAATTCATCGGAGGGAACCAGGTGATC-3’) and crtIhindIIIrev (5’-GGAAGCTTATCCTGCTTTCCGGCCGCGC-3’), respectively. *Eco*RI and *Hin*dIII restriction sites were designed at additional polynucleotides of pucBecoIfor, crtIecoIfor, pucBhindIII and crtIhindIIIrev primers, respectively (underlined). Each fragments cloned in pUC118 vector using *Eco*RI and *Hin*dIII restriction sites. The plasmid obtained called pUCpucB and pUCcrtI were used for making Cy5-labeled DNA probes. Cy5-labeled DNA probes containing either the *pucB*, *crtI* promoter region were amplified by using Cy5-labeled universal primer Cy5-M13for (5’-CAGGAAACAGCTATGAC-3’) and pucBhindIIIrev or crtIhindIIIrev, respectively. Then, the PCR fragment was extracted and purified from 1.5% agarose gel in TAE buffer using QIAquick Gel Extraction Kit (QIAGEN). MT1 and MT2 probes (Fig 1A) were also prepared in the same way using the pUCcrtI changed to MT1 or MT2 sequence by a QuickChange mutagenesis kit.

5 nM DNA probes were incubated for 15 min at room temperature in 7 μl binding reaction buffer composed of 25 mM Tris-HCl (pH 8.0), 100 mM NaCl, 2 mM MgCl_2_, 6% Glycerol and 50 μg/ml heparin. The mixture was incubated with various amounts of purified protein for 30 min at room temperature and then was subjected to 7% polyacrylamide gel electrophoresis at room temperature in a buffer composed of 40 mM Tris-acetate (pH 8.0) and 1 mM ethylenediaminetetracetic acid (EDTA) [17]. After the electrophoresis, the gel was analyzed using the Fluoro Image Analyzer (FUJIFILM, FLA-9000). When reducing conditions was required, fresh dithiothreitol (DTT) of 5 mM final concentration was added into all buffers and the reaction mixture.

### DNase I footprint assay

Fluorescently labeled DNA probes containing either the *pucB* or *crtI* promoter regions were prepared by PCR amplification. 188-bp *pucB* promoter region and 194-bp *crtI* promoter region containing two TGT-N_12_-ACA PpsR-binding palindromes were amplified using a 6-FAM-labeled primer pucBfor (5’-AGAGCTCCCGAGGCCCGC-3’) and a 5-HEX-labeled primer pucBrev (5’-ACCAGTTTCCGTGCTCGACC-3’), a 6-FAM-labeled primer crtIfor (5’-ATCGGAGGGAACCAGGTGAT-3’) and a 5-HEX-labeled primer crtIrev (5’-ATCCTGCTTTCCGGCCGCG-3’), respectively. Then, the PCR fragment was extracted and purified from 1.5% agarose gel in TAE buffer using QIAquick Gel Extraction Kit (QIAGEN).

Individual footprint reactions were initiated with a 22 μl binding reaction mixture that contained 12.5 mM HEPES (pH 7.8), 5 mM K-acetate (pH 8.0), 2.5 mM Mg-acetate, 1 mM CaCl_2_, 12.5 μg/ml bovine serum albumin [18], 0.3 mg/ml heparin, 200 nM of fluorescence-labeled DNA probe and various amounts of purified protein. Initial binding reaction mixtures were incubated for 30 min at 22°C followed by a 15 min DNase I digestion that was initiated by adding 3 μl of DNase I (New England Biolabs) at an approximately 1:100 dilution (0.02 units/μl) in a footprint binding buffer, which gave partial probe digestion. The digestion reactions were then stopped by adding 25 μl of 0.5 M EDTA (pH 8.0). DNA segments were purified using a MinElute PCR purification kit (QIAGEN), with sample eluted with 15 μl of elution buffer containing 0.5 μl of 500 LIZ Size Standard (Applied Biosystems). Samples were then transferred to a 96-well plate [19, 20]. The plate was then sealed using a septum, heated to 95°C for 5 min, and cooled quickly on ice. The samples were separated and detected with a 3730 DNA Analyzer (Applied Biosystems) and analyzed with Peak Scanner Software v1.0 (Applied Biosystems). When reducing conditions was required, fresh DTT was added into all of the buffers and the reaction mixture (10 mM final concentration). Fragment analysis was performed on an Applied Biosystems 3730 automated DNA sequencing machine with fragments detected using the GeneMapper-Generic protocol. Samples were analyzed using SoftGenetics GeneMarker 1.4 analysis software.

## Results

### PpsR-binding sites in photosynthesis gene promoters in *Rvi*. *gelatinosus*

Analysis of the *Rvi*. *gelatinosus* genome sequence (accession number AP012320) indicates the presence of putative PpsR-binding motifs were found in *pucB* and *crtI* promoter regions in *Rvi*. *gelatinosus* and another *Rubrivivax* species *Rvi*. *benzoatilyticus* (Fig 1A). The typical PpsR-binding motif in *Rhodobacter* species is TGT-N_12_-ACA [13], but it is not perfectly conserved in the *Rvi*. *gelatinosus* and *Rvi*. *benzoatilyticus pucB* and *crtI* promoters. Specifically, one of the two PpsR-binding sites, found in the *pucB* promoter, is TGT-N_12_-ACG in the two *Rubrivivax* species. The *crtI* promoter also contains nucleotide variations in the PpsR-binding sites in *Rubrivivax* species as well as in *Rba*. *capsulatus* (Fig 1A). It is also of note that spaces between the two PpsR-binding sites in these promoters are different in these species. Specifically, two PpsR-binding sites in the *pucB* promoter are separated by 9-bp and 240-bp in *Rvi*. *gelatinosus* and *Rba*. *capsulatus*, respectively. In the *crtI* promoters the recognition palindromes are separated by 8-bp and 76-bp in *Rvi*. *gelatinosus* and *Rba*. *capsulatus*, respectively. In this study, we characterized both *pucB* and *crtI* promoters for possible PpsR-binding targets in *Rvi*. *gelatinosus* using *in vivo* and *in vitro* analyses.

### Characterization of Δ*ppsR* and Cys436 to Ala mutants

**Fig 2:**
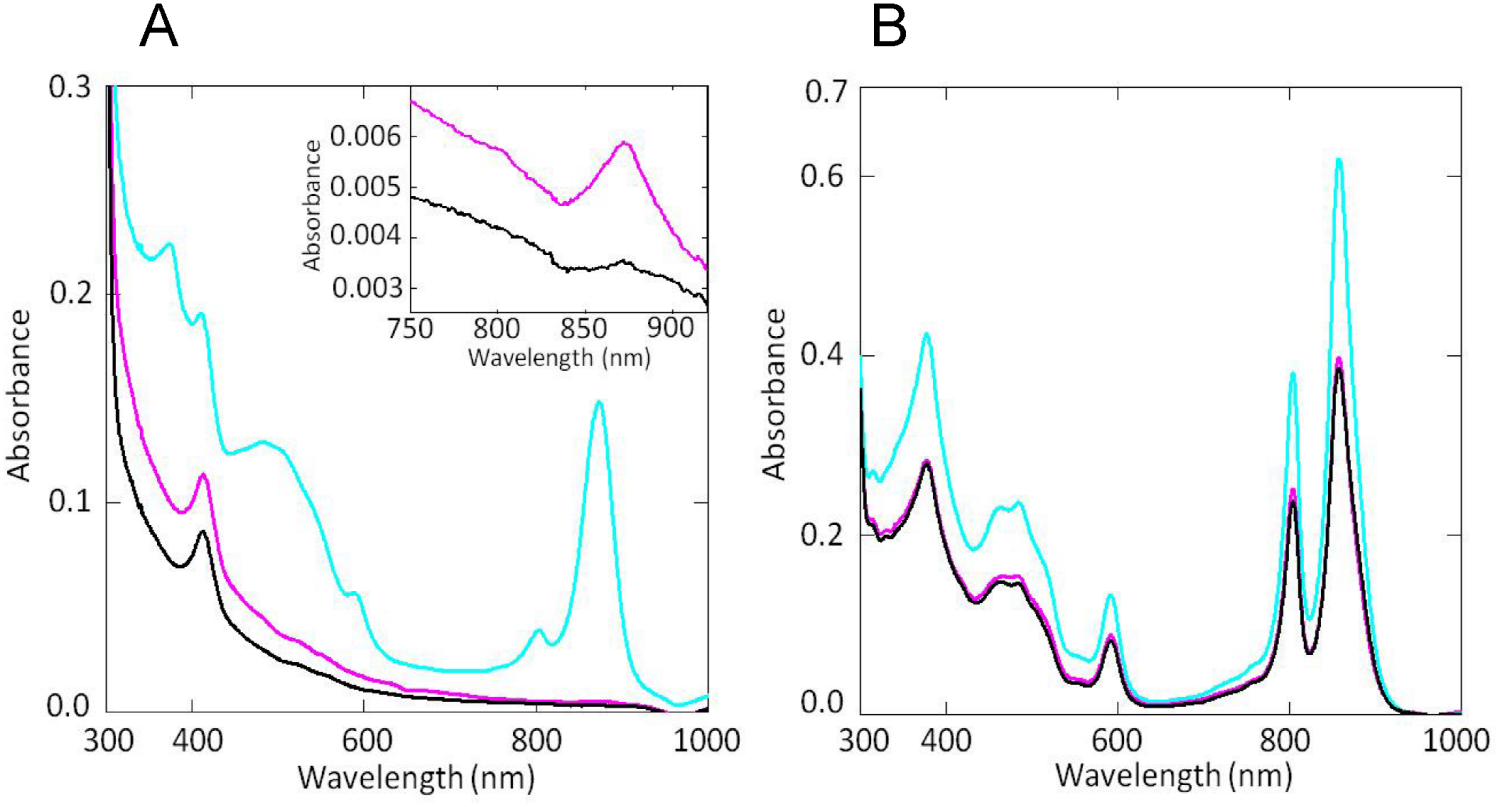
Absorption spectra of membrane fractions of *Rvi*. *gelatinosus* grown under aerobic dark (A) or anaerobic photosynthetic (B) conditions. wild-type (black line), the *ppsR* disrupted mutant (blue line) and the C436A mutant (red line).

A computer-based similarity search indicated that *Rvi*. *gelatinosus* retains only one *ppsR* homolog (RGE_33430) on the genome. We tested if PpsR has an *in vivo* role in controlling photosystem synthesis in *Rvi*. *gelatinosus* by constructing a chromosomal deletion of *ppsR*. We also constructed a strain that had the putative reactive Cys436 present in the HTH domain mutated to Ala. The Δ*ppsR* strain was constructed by a site-specific double recombination event. To avoid the complicating effects on expression of downstream or upstream genes, the clean chromosomal deletion was constructed where the start and stop codon of PpsR were fused (see Materials and methods). The Cys436 to Ala mutation was also constructed using double recombination. Fig 2A and 2B show the result of spectral analysis of *Rvi*. *gelatinosus* mutant strains; Δ*ppsR* strain (blue line) and C436A mutant (red line) and wild-type (black line) under two different growth conditions. This analysis was performed three times, and similar results were obtained. *Rvi*. *gelatinosus* has two types of light harvesting complexes; they are light harvesting (LH) 1 and LH2 which absorb 875 nm and 800–850 nm wavelengths of light, respectively. As shown in Fig 2B, the Δ*ppsR* strain synthesized more LH1 and LH2 complexes than wild type under anaerobic (photosynthetic) conditions. In contrast, C436A mutant showed similar photosynthetic apparatus levels as compared with wild-type in anaerobic growth conditions. When grown aerobically, the Δ*ppsR* strain exhibits significant increased synthesis of LH1 and LH2 as compared to wild-type and C436A mutant (Fig 2A). Interestingly the C436A mutant exhibited levels of the photosynthetic apparatus that are similar to that of wild type with just slightly higher synthesis of LH1 (Fig 2A, inset).

**Fig 3:**
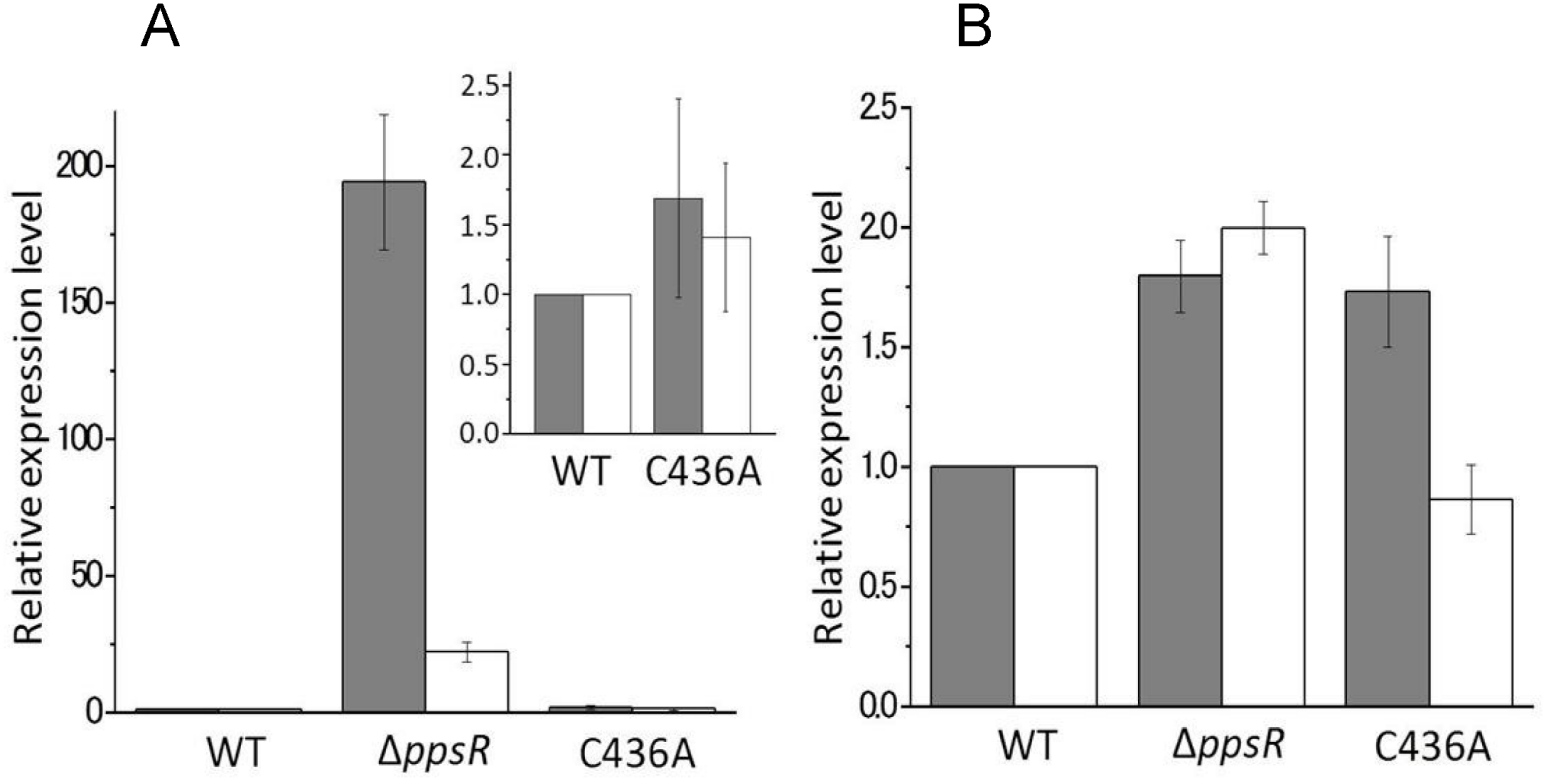
Relative level of transcripts of *pucB* (grey bar) and *crtI* (white bar). Relative expression levels in the *ppsR* disrupted mutant and the C436A mutant as compared with wild-type. Cells were grown under aerobic dark (A) or anaerobic photosynthetic (B) conditions. Error bars represent standard error of the mean (*n* = 3).

We also used quantitative real-time PCR to assess mRNA levels of select individual photosynthesis genes. Under anaerobic conditions, the Δ*ppsR* strain exhibited 1.7- to 2-fold elevated *pucB* and *crtI* expression as compared with wild-type cells (Fig 3B). In contrast, C436A mutant exhibited similar *crtI* expression level when compared to wild-type cells although *pucB* expression was elevated to 1.7-fold (Fig 3B). Under aerobic conditions, the Δ*ppsR* strain exhibited significant up-regulation of *pucB* (~190-fold) and *crtI* (~25-fold) transcription (Fig 3A). C436A mutant also exhibited small (~1.5-fold) but significant elevated *pucB* and *crtI* transcription compared with wild type under aerobic condition (Fig 3A).

### DNA-binding activity of purified *Rvi*. *gelatinosus* PpsR

**Fig 4:**
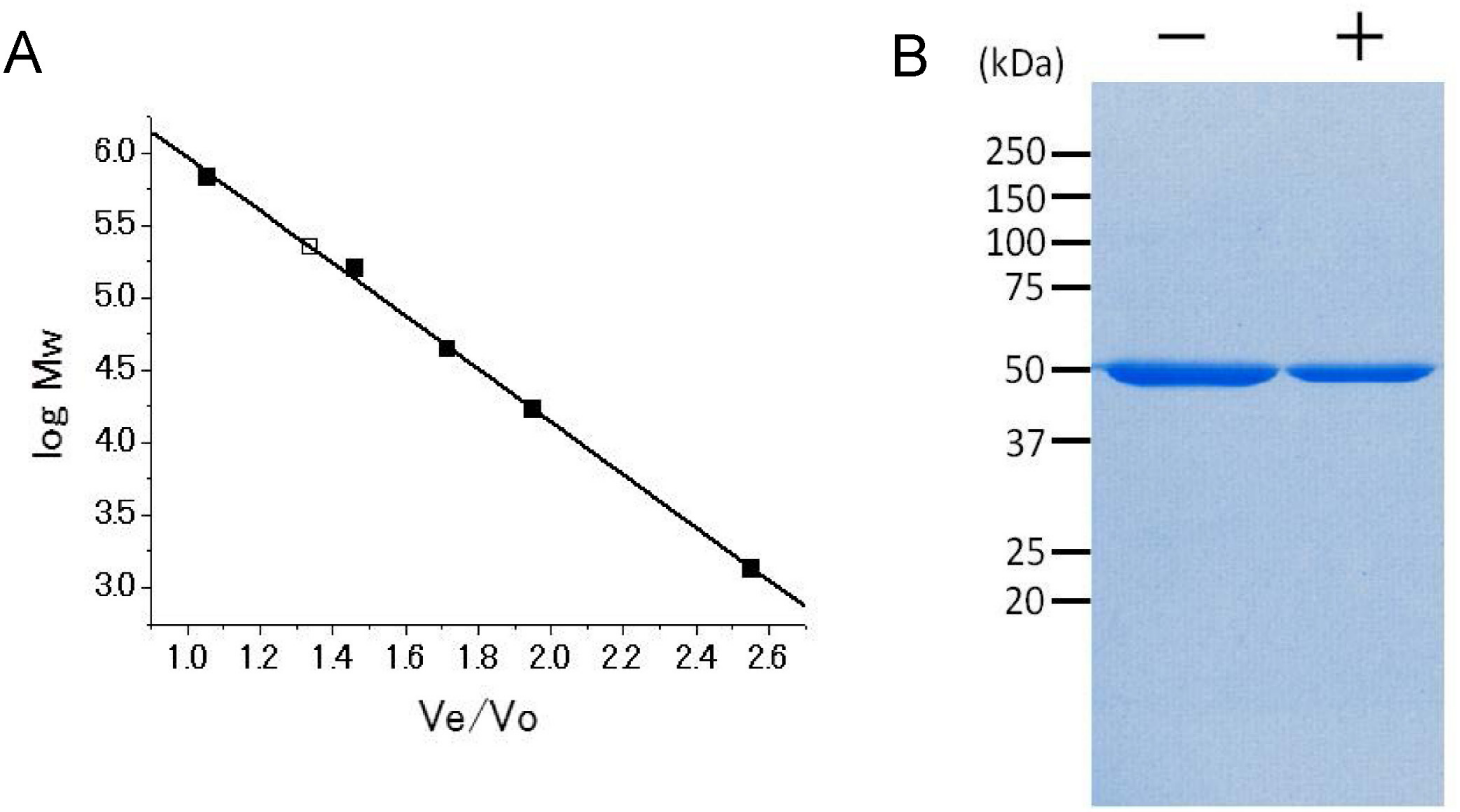
Biochemical properties of *Rvi gelatinosus* PpsR. (A) The molecular weight standard curve of the size exclusion chromatography. The elution points for protein standards and PpsR are indicated by black squares and a white square, respectively. (B) SDS-PAGE analysis of oxidized (left lane) and 5 mM DTT-reduced (right lane) PpsR.

For *in vitro* studies, we purified recombinant *Rvi*. *gelatinosus* PpsR using *E*. *coli* overexpression system followed by affinity chromatography and size exclusion chromatography. The molecular weight of the purified PpsR was estimated to be 223.5 kDa, based on elution profiles of the size exclusion chromatography (Fig 4A). Given the molecular weight of PpsR, calculated from its amino-acid sequence, is 52.4 kDa, the data indicated that PpsR exists as tetramer in solution. Only monomeric 53 kDa band was observed on the SDS-PAGE of the purified PpsR under oxidizing (no treated) and DTT-reducing conditions (Fig 4B), showing that PpsR does not form any intermolecular disulfide bonds.

**Fig 5:**
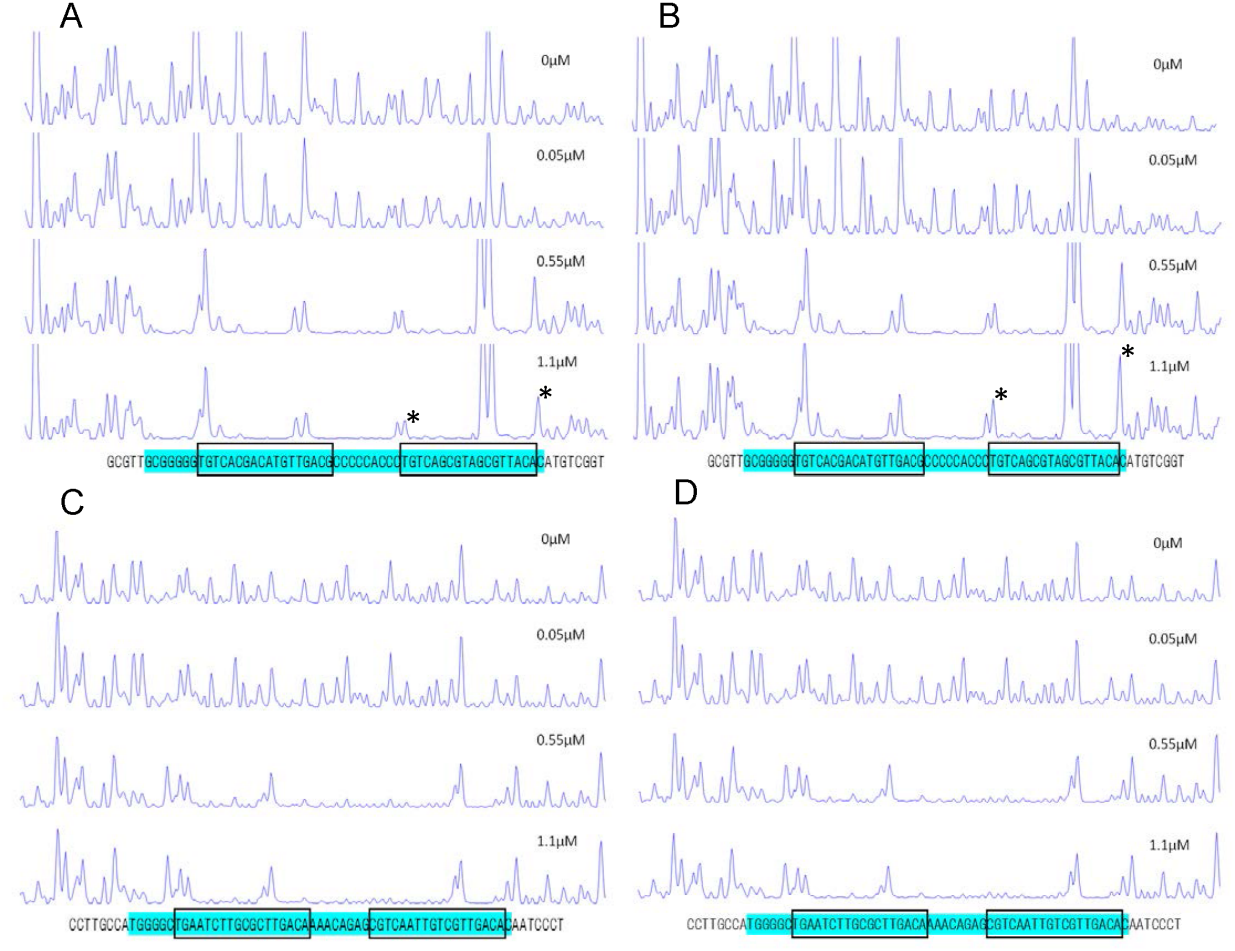
DNase I footprint analysis of PpsR. Binding to the *pucB* promoter region under oxidizing (A) and reducing condition (B), and to the *crtI* promoter region under oxidizing (C) and reducing condition (D). Regions corresponding to the DNase I protection regions are shown in blue background. The possible PpsR-binding sites are boxed letters on the bottom of each figure. Sites for different protection patters observed in oxidized and reduced conditions are indicated with asterisks.

DNase I footprint analysis was undertaken with purified PpsR to assay the extent of protection to the *pucB* and *crtI* promoter regions (Fig 5). The protection pattern showed good protection to both promoter regions centered around the PpsR binding recognition sequences as described above. In the *pucB* promoter region, PpsR binding region overlaps to the -35 and -10 σ-subunit recognition sequences (the promoter recognition sequences in the *crtI* promoter region has not been determined) [21]. These binding patterns are very similar to that reported for the CrtJ/PpsR binding pattern to the *bchC* promoter region in *Rba*. *capsulatus* [22]. Interestingly, neither protection region exhibited large differences between oxidizing (no treated) and reducing (presence of DTT) conditions. The one notable difference is a slight increase in DNase I sensitivity of a nucleotide in the *pucB* promoter region as high-lightened by an asterisks in Fig 5A and 5B.

**Fig 6:**
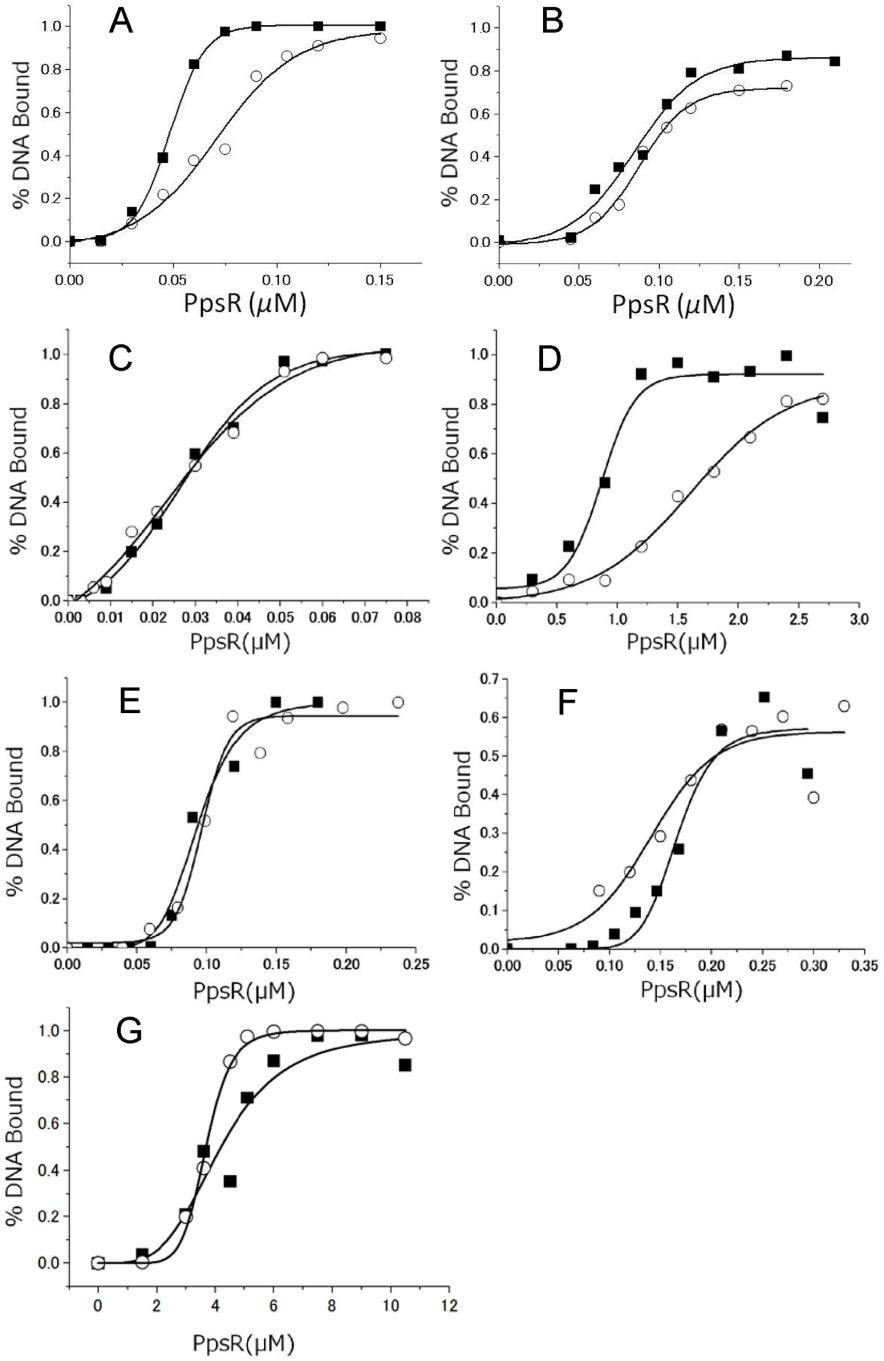
Binding isotherms of oxidized (filled squares) and reduced (open circles) PpsR. DNA-binding percentages were generated by measuring the levels of shifted probes. (A) The binding isotherm of PpsR to the *pucB* promoter region. (B) The binding isotherm of PpsR to the *crtI* promoter region. (C) The binding isotherm of PpsR to the MT1 mutant *crtI* promoter (Fig 2). (D) The binding isotherm of PpsR to the MT2 mutant *crtI* promoter (Fig 2). (E) The binding isotherm of C436A mutant PpsR to the *pucB* promoter region. (F) The binding isotherm of C436A mutant PpsR to *crtI* promoter region. (G) The binding isotherm of C436A mutant PpsR to the MT2 mutant *crtI* promoter. The analysis was performed at least three times, and similar results were obtained, although the binding isotherm of each replicate was shown.

We also assayed the binding affinity by performing gel mobility shift analysis with the purified PpsR and DNA fragments for *pucB* and *crtI* promoters. As shown in Fig 1B and 1C, PpsR clearly binds to both promoters in a concentration-dependent manner. We then performed gel mobility shift analysis with small incremental increases (0.007–0.1 μg) of PpsR to generate binding isotherms for determining EC_50_ values (effective concentration of PpsR for 50% response) of PpsR under oxidizing (no treated) and reducing (in the presence of 5 mM DTT) conditions (Fig 6A–6D). We used no treated PpsR as oxidized PpsR, because purified PpsR is almost oxidized in the absence DTT in *Rhodobacter* species [7]. In fact, purified PpsR of *Rvi*. *gelatinosus* showed similar binding to *pucB* promoter with or without H_2_O_2_ treatment (S1 Fig). The binding affinity of PpsR to the *pucB* promoter under reducing conditions was estimated to be EC_50_ = 63.26 nM which was a modest 1.27-fold larger than that under oxidizing conditions (49.82 nM) (Fig 6A, Table 1). Furthermore, the binding curve under oxidizing conditions showed more sigmoidal than under reducing conditions (Fig 6A). On the other hand, binding affinity of PpsR to the *crtI* promoter region showed no significant difference between reducing (EC_50_ = 86.46 nM) and oxidizing (EC_50_ = 80.46 nM) conditions (Fig 6B, Table 1). It should be noted that the binding affinity of PpsR to the *crtI* promoter region was lower than that observed for the *pucB* promoter region (Table 1).

**Table 1:** Binding affinity of WT and the C436A mutant of PpsR to *pucB*, *crtI*, *crtI*_MT1 and *crtI*_MT2 promoter DNA probes under oxidizing and reducing conditions.

DNA Probes	Conditions	EC_50_ (nM)	
		WT	C436A
*pucB*	oxidized	49.8 (± 2.3)^a^	94.1 (± 1.9)
	reduced	63.3 (± 4.1) ^a^	93.3 (± 7.0)
*crtI*	oxidized	80.5 (± 4.4)	146.4 (± 17.9)
	reduced	86.5 (± 7.2)	146.6 (± 3.4)
*crtI*_MT1	oxidized	30.8 (± 1.4)	NT
	reduced	31.9 (± 2.9)	NT
*crtI*_MT2	oxidized	972.6 (± 159.0)^b^	4646.3 (± 848.4)
	reduced	1478.5 (± 155.9) ^b^	4515.1 (± 416.4)

^a^ *P* < 0.05, *t*-test (compared each other).

^b^ *P* < 0.05, *t*-test (compared each other).

We next constructed two variants of the *crtI* promoter (MT1 and MT2) that exhibited a sequence structures that are more typical of previously characterized PpsR-binding sites (TGT-N_12_-ACA). For probe MT1 the *Rvi*. *gelatinosus* two PpsR recognition sequences in the *crtI* promoter was changed from [(TGa-N_12_-ACA)-N_8_-(cGT-N_12_-ACA)] to [(TGT-N_12_-ACA)-N_8_-(TGT-N_12_-ACA)]. This probe thus contains a PpsR binding site with the caveat that the spacing between the two TGa-N_12_-ACA recognition sequences is 8 instead of the 9 nucleotides. For probe MT2, the two divergent PpsR recognition sequences remained as present in the wild sequence with the caveat that the spacing between the two recognition sequences was changed from 8 to 9 nucleotides as in the *pucB* promoter (Fig 1A). The results of gel mobility shift analysis indicates that the binding affinity of PpsR to the MT1 probe [(TGT-N_12_-ACA)-N_8_-(TGT-N_12_-ACA)] was higher than that to native promoter probe. Furthermore as is the case of the wild type probe, there was no significant difference in binding observed between reducing (EC_50_ = 31.89 nM) and oxidizing conditions (EC_50_ = 30.81 nM) (Fig 6C, Table 1) with the MT1 probe. On the other hand, the binding affinity of PpsR to the MT2 probe that contains the 9-bp-spacing between the degenerate recognition sequences was significantly higher under oxidizing conditions (EC_50_ = 972.02 nM) than under reducing conditions (EC_50_ = 1478.50 nM) (Fig 6D, Table 1). Furthermore, the binding curve under oxidizing conditions changed into more sigmoidal than under reducing conditions (Fig 6D).

We also measured the binding affinity of PpsR C436A mutant in which the conserved putative redox responding Cys in the HTH domain was replaced with Ala (Fig 6E–6G). The binding affinity of PpsR C436A to the *pucB* promoter region was estimated to be EC_50_ = 94.13 nM and 93.25 nM under oxidizing and reducing conditions, respectively (Fig 6E, Table 1). To the *crtI* promoter region, the binding affinity was estimated to be EC_50_ = 146.36 nM and 146.59 nM under oxidizing and reducing conditions, respectively (Fig 6F, Table 1). Thus, EC_50_ values for C436A mutant PpsR to the wild type *pucB* and *crtI* promoter regions are no significant difference between oxidizing and reducing conditions. Furthermore, there was also no significant difference in binding observed between oxidizing (EC_50_ = 4515.14 nM) and reducing conditions (EC_50_ = 4646.31 nM) (Fig 6G, Table 1) with the MT2 probe.

## Discussion

Whole cell analysis of photopigment accumulation shows that PpsR has an important role in controlling photosystem synthesis in *Rvi*. *gelatinosus* not unlike that observed in *Rhodobacter* species. This conclusion is based on the observation that pigment accumulation was significantly larger in the aerobically grown Δ*ppsR* strain than in wild type *Rvi*. *gelatinosus* (Fig 2A). The transcript levels of *pucB* and *crtI* in the aerobically grown Δ*ppsR* strain were also higher than that observed in wild type cells (Fig 3A). These results suggest that PpsR in *Rvi*. *gelatinosus* acts as a repressor of photosynthesis gene expression under both aerobic and anaerobic conditions. This is quite different from that observed with PpsR homologs from *Rhodobacter* species where they primarily function as aerobic repressors [4, 5]. PpsR function in *Rvi*. *gelatinosus* as an semiaerobic repressor and activator has also been reported [23]. What is less clear is the mechanism of controlling the DNA binding activity of PpsR in this α-proteobacterium. In *Rhodobacter* species, mutation of a conserved Cys located in the HTH domain of PpsR/CrtJ results in a disruption of redox sensing [9]. However, an equivalent Cys436Ala mutant of *Rvi*. *gelatinosus* PpsR has almost no discernable phenotype *in vivo* (Figs 2 and 3). This suggests that Cys436Ala may have a minimal role in the control of PpsR activity *in vivo*. Assuming that PpsR has only minor redox control in this species, then PpsR may control photosystem gene expression under both aerobic and anaerobic conditions with inputs other than redox regulating its activity. In *Rba*. *sphaeroides* it has been shown that PpsR is controlled by the redox state of a conserved Cys in the HTH domain as well as by the antirepressors AppA and PpsA [24, 10]. The antirepressor activity of the AppA is controlled by light excitation of a bound flavin as well as the presence of heme [7]. In *Rba*. *capsulatus*, it was recently shown that a PpaA homolog called AerR controls the DNA binding activity of the PpsR homolog CrtJ in response to light-dependent binding of cobalamin (vitamin B_12_) [25]. The characterization of a potential antirepressor of *Rvi*. *gelatinosus* PpsR is beyond the scope of this study, but our *in vivo* results indicate regulation of PpsR activity other than the redox state of Cys436 is likely very important in this species.

Our *in vivo* results are also supported by several of our *in vitro* studies with isolated PpsR. Specifically, we observed excellent DNase I footprint protection of PpsR to the *puc* and *crtI* promoter regions under both oxidizing and reducing conditions (Fig 5). The only observed difference was a minor enhancement of a single peak in the *puc* promoter under reducing conditions. Gel mobility shift results also shown that the binding affinity of *Rvi*. *gelatinosus* PpsR to the *pucB* promoter was only slightly higher (1.3-fold) under oxidizing conditions than under reducing conditions (Table 1). This minor effect is in stark contrast to *Rhodobacter* PpsR which exhibits a 19-fold difference in redox control *in vitro* [8]. Cys436 does appear to have a role in this minor redox control as the binding affinity of the Cys436Ala mutant PpsR to the *pucB* promoter region showed no significant difference between oxidizing and reducing conditions (Table 1).

Interestingly the binding affinity of wild type PpsR to the *crtI* promoter region showed no significant difference between oxidizing and reducing conditions (Table 1). When the space between the two *crtI* PpsR-binding sites was changed to 9-bp, the binding of PpsR to this site gained redox control as evidence by higher binding under oxidizing conditions than under reducing conditions (Table 1). Furthermore, the binding affinity of PpsR C436A to this changed promoter showed no significant difference between under oxidizing and reducing conditions (Table 1). Collectively these results indicate that PpsR can modestly affects DNA binding based on the redox state of the conserved Cys436. However, this modest level of redox control is apparently only operative when the space between the two PpsR-binding sites 9-bp than 8-bp. Interestingly, the binding curve of PpsR to the *crtI* promoter is more sigmoidal indicating more cooperative when the spacing is 9-bp than when it is 8-bp. This indicates that cooperative binding of PpsR to its target sequence is dependent on appropriate spacing between the PpsR binding sites and that the observed redox control may be affecting such cooperative interactions. Finally, in another *Rubrivivax* species (*Rvi*. *benzoatilyticus*), spaces between the two PpsR-binding sites in the *pucB* and *crtI* promoters are the same as those in *Rvi*. *gelatinosus*, specifically 9 vs 8-bp (Fig 1A). Therefore, different spacing in the *pucB* and *crtI* promoters may be characteristics to *Rubrivivax* species. Why would the binding affinity of *Rvi*. *gelatinosus* PpsR differ between *pucB* and *crtI* promoters? If photosystems are synthesized in the presence of oxygen, then light excitation of bacteriochlorophylls would generate ROS. In order to avoid production of ROS, *puc* genes encoding LH complex apo-proteins, seem to be repressed under aerobic conditions. In contrast, carotenoids themselves work as a ROS scavengers where they can decay reactive singlet oxygen back to its ground state by absorbing energy from singlet oxygen [26]. Therefore, it is possible that carotenoid synthesis is by design not repressed under aerobic conditions. The lower affinity of *Rvi*. *gelatinosus* PpsR to *crtI* promoter region than *pucB* promoter region (Table 1) might indicate such necessity of carotenoid production under aerobic conditions. The reason for repression of carotenoid synthesis in *Rhodobacter* species under aerobic conditions might be related to their more enhanced capability to repress synthesis of their photosynthetic apparatus in response to oxygen.

β-proteobacteria are thought to have obtained a photosynthesis gene cluster by horizontal gene transfer from α-proteobacteria [14, 27]. Thus, *Rvi*. *gelatinosus* might have adapted their regulatory genes to coordinate photosynthesis and respiration that is unique to this species. Cleary, PpsR orthologs in purple bacteria have species specificity in their redox sensing mechanisms that differ from that described for the *Rhodobacter* paradigm.

## Supporting Information

S1 Fig Binding isotherms of no treated (filled squares) and H_2_O_2_ treated (filled triangle) PpsR. The binding isotherm of PpsR to the *pucB* promoter region. For H_2_O_2_ treatment, PpsR was incubated with five times molar concentration of H_2_O_2_ for an hour at room temperature.
(TIF)
