# Supplementary figures and images for: Evidence that Altered Cis Element Spacing Affects PpsR Mediated Redox Control of Photosynthesis Gene Expression in Rubrivivax gelatinosus
Source: PLoS One. 2015 Jun 1;10(6):e0128446. doi: 10.1371/journal.pone.0128446 (PMC4452267; doi:10.1371/journal.pone.0128446)

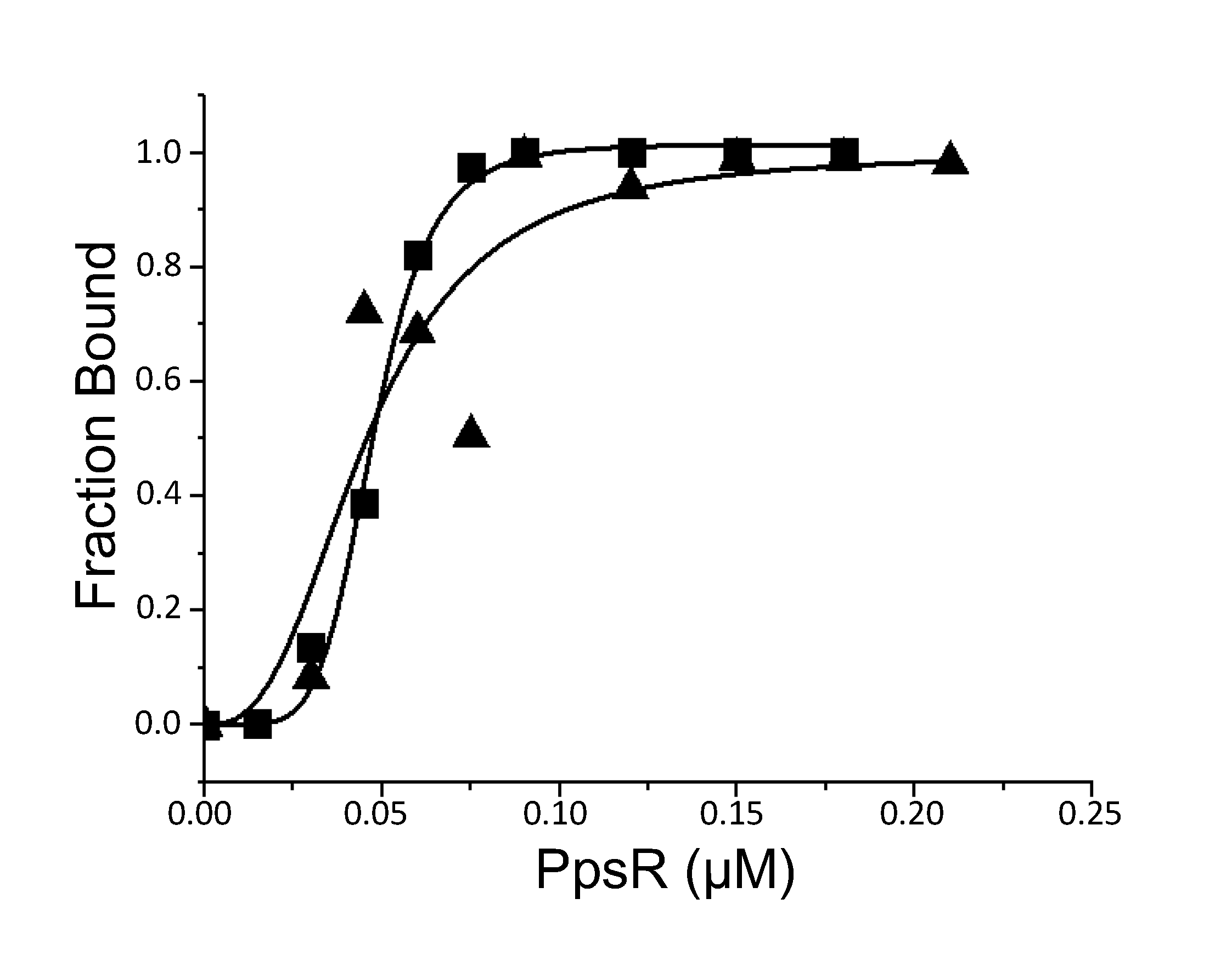

Supplement: S1 Fig — The binding isotherm of PpsR to the pucB promoter region. For H2O2 treatment, PpsR was incubated with five times molar concentration of H2O2 for an hour at room temperature. (TIF) [file pone.0128446.s001.tif]
